# 表现为脑钙化灶的肺癌脑及脑膜转移1例报告并文献复习

**DOI:** 10.3779/j.issn.1009-3419.2025.106.05

**Published:** 2025-03-20

**Authors:** Deng ZHANG, Yiru KONG, Xiaohua LIANG, Xinli ZHOU

**Affiliations:** 200040 上海，复旦大学附属华山医院肿瘤科; Department of Oncology, Huashan Hospital, Fudan University, Shanghai 200040, China

**Keywords:** 肺肿瘤, 脑转移, 脑膜转移, 钙化, 计算机断层扫描, 磁共振成像, Lung neoplasms, Brain metastases, Meningeal metastases, Calcification, CT, MRI

## Abstract

肺癌目前仍是全球最常见的恶性肿瘤之一，随着其发病率的升高及医疗技术的发展，肺癌患者的总生存期较前明显延长。肺癌脑及脑膜转移发生率也逐年升高，但预后较差，病死率极高，诊断主要通过计算机断层扫描（computed tomography, CT）及磁共振成像（magnetic resonance imaging, MRI）等影像学检查，但影像特征多样，特异性较低，极易误诊及漏诊。因此，准确识别脑及脑膜转移并及时进行针对性治疗，对于改善肺癌患者的预后至关重要。本文通过对1例肺癌根治术后近7年长期随访无明显复发转移但近5个月患者出现行为异常、意识障碍及癫痫等症状，头颅CT及MRI发现脑部多发点状钙化灶病例的诊疗经过进行分析，在排除感染及自身免疫性脑炎相关治疗无效后，考虑患者精神行为症状是肺癌脑及脑膜转移所致，进一步病理活检及基因检测，明确诊断为转移性肺腺癌且表皮生长因子受体（epidermal growth factor receptor, EGFR）L858R基因突变，使用奥希替尼靶向治疗后患者症状明显改善；同时，检索中国知网、万方、UpToDate、PubMed等数据库关于脑部钙化灶的相关文献并进行分析，发现脑内钙化存在于多种疾病中，包括感染性、遗传性和神经退行性疾病、血管相关综合征、代谢性疾病和肿瘤，但钙化相关的脑（膜）转移灶往往是被低估的，随之而来的风险是误诊和延迟治疗。因此，对于既往罹患肿瘤病史的患者，以钙化为主要表现的脑（膜）转移灶不容忽视。

## Abstract (en)

Lung cancer is still one of the most common malignant tumors in the world. With the increase of its incidence and the development of medical technology, the overall survival of lung cancer patients has significantly extended compared to before. The incidence of brain and meningeal metastases from lung cancer has also been rising year by year, but patients with brain and meningeal metastases from lung cancer have a poor prognosis and a very high mortality rate, and the diagnosis is mainly based on computed tomography (CT), magnetic resonance imaging (MRI) and other imaging examinations. However, the imaging features are diverse and the specificity is low, which makes it easy to be misdiagnosed and missed. Therefore, accurately identifying brain and meningeal metastases and timely targeted treatment is crucial for improving patient prognosis. This paper analyzed the diagnosis and treatment of a case of lung cancer with no obvious recurrence and metastasis in nearly 7-year long-term follow-up after radical lung cancer surgery, but the patient with abnormal behavior, impaired consciousness and epilepsy in the past 5 months, and multiple punctate calcifications in the brain found by head CT and MRI. This paper consider that the patient's mental and behavioral symptoms were caused by brain and meningeal metastasis of lung cancer after excluding infectious disease and ineffective treatment of autoimmune encephalitis, and further pathological biopsy and genetic detection confirmed the diagnosis of metastatic lung adenocarcinoma with epidermal growth factor receptor (EGFR) L858R gene mutation, and the patient's symptoms were significantly improved after targeted therapy by Osimertinib. This paper also searched the relevant literatures of brain calcifications in databases such as China National Knowledge Infrastructure (CNKI), Wanfang, UpToDate, PubMed, etc., and found that intracerebral calcifications exist in a variety of diseases, including infectious, genetic and neurodegenerative diseases, vascular diseases, metabolic diseases and tumors. However, brain calcification in brain and meningeal metastases are often underestimated, and the consequent risk is misdiagnosis and delayed treatment. Therefore, brain and meningeal metastases manifested as brain calcification should not be ignored in patients with a history of previous tumors.

肺癌是恶性肿瘤患者中死亡率最高的疾病^[1]^，其发病率及死亡率逐年上升，对人类健康和生命构成严重威胁。其中，非小细胞肺癌（non-small cell lung cancer, NSCLC）约占肺癌的85%^[2]^，主要病理组织类型包括肺腺癌和鳞状细胞癌等，晚期NSCLC患者容易发生脑转移，主要包括脑实质转移和软脑膜转移^[3]^。其中，肺癌脑实质转移是指肺来源的恶性肿瘤细胞通过血液循环或淋巴系统扩散至脑组织内形成的病变，肺癌脑膜转移是肺来源的恶性肿瘤细胞在脑膜或脑脊液中发生扩散^[4]^。随着医疗技术的发展，肺癌患者的生存期较前明显延长，晚期肺癌合并脑和/或脑膜转移的发生率显著升高。发生肺癌脑和/或脑膜转移的患者预后不良，病死率极高^[5]^，但肺癌脑及脑膜转移临床表现缺乏特异性，主要应用计算机断层扫描（computed tomography, CT）及磁共振成像（magnetic resonance imaging, MRI）等检查进行诊断，而脑及脑膜转移影像学特征多样，通常表现为环形强化、边缘清晰的强化病灶、低密度区无明显强化及混杂密度病灶等。这些影像特征与其他颅内病变（如脑膜瘤、脑脓肿等）极为相似，易导致误诊及漏诊。因此，准确识别脑膜转移及脑转移瘤并及时进行针对性治疗，对于改善患者预后至关重要。本文报道1例表现为脑钙化灶的肺癌脑及脑膜转移病例（本病例报道的发表已获得患者及家属的知情同意，并经复旦大学附属华山医院的伦理审查委员会批准同意发表），同时检索中国知网、万方、UpToDate、PubMed等数据库关于脑部钙化灶的相关文献并进行分析探讨。

## 1 病例资料

患者，女，67岁，因“言语行为异常5月余，意识障碍4天”于2024年7月1日入院。患者于2024年2月左右开始出现言语行为异常，初始自言自语，偶有脾气暴躁易怒等性格改变，后症状逐渐加重，无法正常交流。2024年3月患者洗澡穿衣等生活自理能力逐渐丧失，就诊于当地医院，3月14日头颅MRI显示右侧额叶及双侧颞叶皮层多发钙化灶，颜面部血管瘤病待排。弥散加权成像（diffusion weighted imaging, DWI）显示两侧侧脑室旁、两额顶叶白质区散在血管源性白质高信号可能，Fazekas 2级，两侧脑白质疏松，空泡蝶鞍。3月25日行腰椎穿刺术，脑脊液常规、生化未见明显异常，脑脊液和血自身免疫性脑炎抗体谱及副肿瘤综合征抗体谱未见明显异常。当地医院给予奥氮平（再普乐）、劳拉西泮（佳普乐）、盐酸美金刚（易倍申）等对症治疗后患者症状仍逐渐加重。4月1日行正电子发射计算机断层显影（positron emission tomography/CT, PET/CT）显示双侧额叶（右侧为著）、双侧顶叶、双侧颞叶多发点片状钙化灶，伴糖代谢降低，左侧小脑糖代谢减低，交叉失联络可能。患者于2024年4月10日就诊于复旦大学附属华山医院，尚可行走，偶有小便失禁，但对答不能。入院后完善腰椎穿刺术，脑脊液常规：颜色：无色，透明度：清，白细胞计数（white blood cell, WBC）为6×10^6^/L，红细胞计数（red blood cell, RBC）为1×10^6^/L，潘氏试验为阴性。脑脊液生化：糖为2.28 mmol/L（↓），氯为125 mmol/L，蛋白为553 mg/L，乳酸为1.85 mmol/L，乳酸脱氢酶为76.00 U/L。血+脑脊液自身免疫性脑炎抗体10项检查，血副肿瘤综合征抗体22项阴性，血清组织分析（tissue based assay, TBA）抗体阳性，脑脊液TBA抗体阴性，考虑为自身免疫性脑炎，予以激素序贯冲击治疗并减量至口服，患者症状未见明显改善。后予以5次血浆置换治疗，并分别于4月27日和4月28日予以利妥昔单抗100及500 mg免疫治疗，出院后继续口服激素治疗，患者言语行为异常较前无明显改善。患者于6月27日出现左侧口角抽动，随即左侧肢体抽搐至全身抽搐，持续约3 h，至当地医院就诊给予镇静药后抽搐较前好转，仍间断有左手抽动，左侧肢体活动较差。患者于6月29日就诊于复旦大学附属华山医院急诊，头颅CT（图1）显示右侧额颞顶叶及左侧颞叶脑回多发钙化，双侧额顶叶及侧脑室周围多发缺血腔隙灶，轻度脑萎缩，建议MRI检查。给予奥卡西平（万仪）、丙戊酸钠缓释片（喜复至）抗癫痫发作，患者肢体抽搐较前缓解，意识状态好转，左侧肢体活动有所好转，但仍完全性失语。2024年7月1日为进一步查找病因收入复旦大学附属华山医院。既往史：患者2017年发现肺腺癌（T1N0M0 I期）行手术根治，无需进一步放化疗，未进一步完善肿瘤组织基因检测。后患者定期复查，在2022年12月左右患者头颅CT未见明显异常及钙化灶。

**图1:**
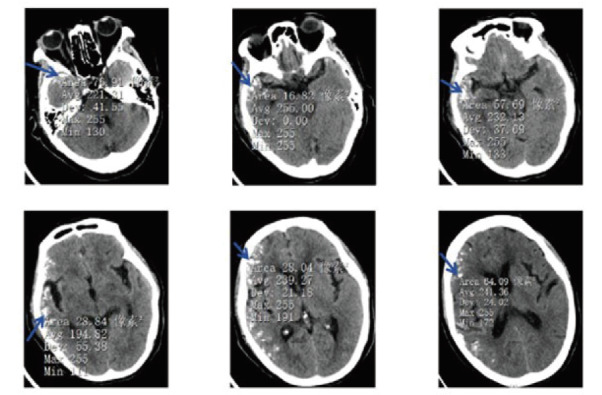
CT上不同层面的颅内钙化灶（平均CT值：194-255 Hu）

2024年7月1日患者入院后进行实验室检查和影像学检查：（1）血常规：WBC为4.49×10^9^/L，RBC为4.49×10^12^/L，血红蛋白（hemoglobin, Hb）为127 g/L，C反应蛋白（C-reactive protein, CRP）为5.76 mg/L；（2）D-二聚体（D-dimer, D-D）为9.35 mg/L，尿常规、粪常规、血生化等未见明显异常；（3）肿瘤标志物癌胚抗原（carcinoembryonic antigen, CEA）为38.7 ng/mL（↑），余未见明显异常；（4）7月3日行腰椎穿刺术，脑脊液压力为200 cm H_2_O，脑脊液常规：颜色：无色，透明度：清，WBC为8×10^6^/L，RBC为1×10^6^/L，潘氏试验：阴性；脑脊液生化：糖为3.03 mmol/L，氯为118 mmol/L，蛋白为464 mg/L；（5）7月4日头颅增强MRI（图2）显示右侧额顶颞叶及左侧颞叶皮层多发点片状异常强化灶，结合CT相应部位脑回多发钙化表现需考虑脑颜面血管瘤病，两侧额顶叶及侧脑室旁多发缺血灶，轻度脑萎缩；（6）7月8日脑脊液细胞学检查：有核细胞明显增生，以单核组织巨噬细胞为主，寻找可见少量异型细胞（图3）。

**图2:**
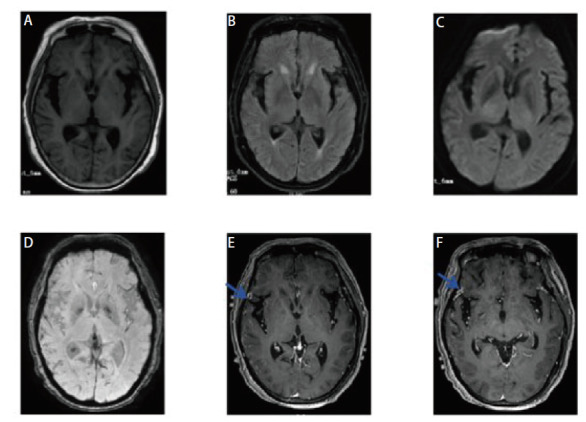
头颅MRI增强不同序列上的脑钙化灶转移瘤表现。A：T1W低信号；B：T2 Flair高信号；C：DWI无明显异常；D：SWI上多发钙化灶；E，F：T1增强序列上右侧额顶颞叶及左侧颞叶皮层多发点片状异常强化灶。

**图3:**
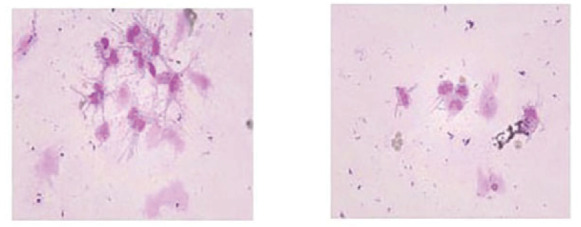
脑脊液中异型细胞（Wright染色，×400）

结合患者认知下降、癫痫发作及头颅影像学典型表现，符合脑颜面血管瘤病，其特征表现是面部酒红色痣和软脑膜血管瘤，多数患者为一侧头面部受累，95%以上患者有面部血管痣，主要症状为自幼开始的癫痫发作。但该例患者面部无血管痣，且既往无癫痫病史，患者2022年12月的头颅CT未见明显颅内钙化灶，短期颅内形成钙化不符合脑颜面血管瘤病。因此，已排除感染，按自身免疫性脑炎治疗无效、既往7年前有肺癌手术史、目前血CEA升高，患者脑脊液细胞学可见少量异型细胞，考虑其肺癌脑（膜）转移的可能性大。7月12日行脑组织活检手术可见，脑膜及皮层即见异常病灶，黄白色，质地偏韧，血供中等，含颗粒状钙化成分，冰冻见异型细胞。7月22日病理报告：（右颞）少量组织，结合免疫组化及病史考虑转移性肺腺癌。免疫组化结果：细胞角蛋白（cytokeratin, CK）（+），细胞角蛋白7（cytokeratin 7, CK7）（+），甲状腺转录因子1（thyroid transcription factor-1, TTF-1）（+），新天冬氨酸蛋白酶A（new aspartic proteinase A, NapsinA）（+），肿瘤增殖标志物Ki-67（marker of proliferation Ki-67, Ki-67）（约3%），嗜铬粒蛋白A（chromogranin A, CgA）（-），突触素（synapsin, Syn）（-），胶质纤维酸性蛋白（glial fibrillary acidic protein, GFAP）（-），上皮膜抗原（epithelial membrane antigen, EMA）（-），孕激素受体（progesterone receptor, PR）（-），生长抑素受体2a（somatostatin receptor 2a, SSTR2a）（-）。CK7、TTF-1、NapsinA阳性主要见于肺腺癌，CgA、Syn阴性暂不考虑神经内分泌肿瘤，Ki-67约3%表明肿瘤细胞增殖相对缓慢，肿瘤侵袭性和转移能力相对较弱。脑活检钙化组织基因检测：表皮生长因子受体（epidermal growth factor receptor, EGFR）p.L858R基因突变（表1）。后续予奥希替尼靶向治疗1个月左右后，患者精神状态、认知能力较前明显好转，能简单交流。后续出院后继续服用靶向药，门诊随访。

**表1:** 脑组织基因检测报告（具有明确临床意义的基因变异）

Gene/Transcript number	Variation information	Type of variation	Proportion of variation/Number of copies	Targeted drug tips (susceptible/drug-resistant, evidence grade)
EGFR NM_005228.5	c.2573T>G p.L858R exon21	Missense mutation	34.48%	Erlotinib (susceptible, A)
				Erlotinib+Ramucirumab (susceptible, A)
				Gefitinib (susceptible, A)
				Osimertinib (susceptible, A)
				Dacomitinib (susceptible, A)
				Afatinib (susceptible, A)
				Furmonertinib (susceptible, A)
				Ivonescimab (susceptible, A)
				Sintilimab+Bevacizumab (susceptible, A)
				Icotinib (susceptible, A)
				Befotertinib (susceptible, A)
				Almonertinib (susceptible, A)
				Amivantamab-vmjw (susceptible, A)
				Erlotinib+Bevacizumab (susceptible, A)
				Gefitinib+Apatinib (potentially susceptible, C)

EGFR: epidermal growth factor receptor.

## 2 讨论

颅内钙化包括生理性/年龄相关的钙化和广泛的病理性钙化^[6]^。生理性钙化常是CT扫描的偶然发现，在老年人中更为普遍，通常见于松果体、脉络膜丛、大脑镰和小脑幕等，其中以松果体钙化最为常见，生理性钙化斑点对脑功能无明显影响^[7]^。病理性钙化可见于脑部的感染性病变、慢性炎症、肿瘤、脑血管硬化及某些脑部退行性病变等^[8,9]^，患者可能表现出神经和/或精神症状，发病年龄和严重程度各不相同，临床表现包括运动障碍，如帕金森综合征、震颤、舞蹈病、抽搐、多动性缄默症和共济失调等。与脑钙化相关的其他神经系统症状包括癫痫发作或卒中事件，以及各种认知和精神症状，如痴呆、精神病、情绪和其他行为障碍等^[10]^。不同类型的疾病其颅内钙化灶分布和形态不同，如：（1）感染性疾病：①病毒感染：巨细胞病毒感染相关的颅内钙化主要常见于脑室周围区、脑实质或基底节区，脑室周围钙化通常表现为厚块状，而基底节区钙化通常为小点状；单纯疱疹病毒性脑炎（图4）钙化主要分布在皮层，多呈脑回状分布^[11]^。②结核感染（图5）：钙化灶主要分布在脑池或脑沟裂邻近部位，以颅底、鞍区等部位多见，钙化常呈不规则斑片状或结节状，结核瘤可能在中心钙化，脑膜钙化不太常见^[12]^。③寄生虫感染：脑囊虫病（图6）可见大脑半球散在、大小相似的环形强化病变，伴有邻近水肿，其特征表现是“靶征”，周围钙化囊肿内表现为偏心钙化结节^[13]^；脑包虫病可见囊壁上完整或不完整的壳状钙化；弓形虫感染表现在脑室周围区和皮层的钙化呈结节状，而丘脑和基底节区的钙化呈曲线状。（2）遗传或发育障碍相关：如Sturge-Weber综合征，即脑三叉神经血管瘤病，特征是面部酒红色痣，同侧软脑膜血管畸形和颅内钙化，表现为局限性脑回状或花纹状钙化，一般为单侧性^[14]^。（3）颅内血管钙化：血管钙化出现在高达90%的动脉粥样硬化病变中，也见于海绵状血管瘤、动静脉畸形、硬脑膜动静脉瘘和动脉瘤等疾病中^[15]^。（4）代谢性疾病：甲状旁腺功能减退症、甲状旁腺功能亢进症等，钙化最显著出现在双侧基底神经节和苍白球，也可能发生在齿状核、皮层下白质和丘脑，常呈双侧对称性钙化，钙化面积较大^[16]^。（5）Fahr病（图7），又称家族性特发性基底节钙化，该病是一种罕见的神经退行性疾病，其双侧苍白球、壳核、丘脑、小脑齿状核呈大片对称性钙化，双侧脑室旁呈放射状钙化，皮髓质交界处点状、线状钙化；钙化由少到多呈进行性发展^[16,17]^。（6）炎症性疾病：如系统性红斑狼疮，其钙化是双侧对称的，最常累及小脑，其次是苍白球、壳核、尾状核和丘脑^[18]^。（7）肿瘤性疾病：颅内钙化被认为是鉴别和评价脑肿瘤的重要工具，钙化的存在与否与患者的年龄和肿瘤的类型与位置有关。颅内原发肿瘤中少突胶质细胞瘤（图8）钙化最常见，成人中可达90%，其钙化与肿瘤内血管相关，可延伸至周围脑实质^[19]^，呈结节状和团块状等。出现钙化的脑转移瘤其原发性肿瘤有肺鳞状细胞癌和腺癌、乳腺癌、纵隔肉瘤、宫颈鳞状细胞癌、胰腺癌、非霍奇金淋巴瘤、骨肉瘤以及结直肠癌和卵巢腺癌等^[20⇓-22]^，其中以源自肺癌和乳腺癌的转移瘤钙化相对多见^[23,24]^，但总体脑转移瘤钙化的发生较为少见，仅在约1%的手术标本中发现^[25]^。此外，脑转移瘤钙化的发生主要见于接受放疗的患者^[12,23,26]^。在既往的病例报道^[27]^中描述了关于脑转移瘤内钙化的不同类型，如点状、曲线状和无定形钙化等，大多数脑转移瘤钙化是点状的，钙化类型与原发肿瘤的组织学之间没有特异相关性。因此，在颅内钙化的鉴别诊断中，脑转移瘤钙化可能会被低估^[28]^，该例患者仅有7年前早期肺癌手术史，无脑部放疗史，病程中颅外一直无复发转移迹象，最终依赖手术活检脑部钙化灶方确诊为肺癌脑转移，此时距离患者以精神异常起病已达半年。综上所述，针对具有恶性肿瘤病史的患者，在影像学上观察到脑内钙化灶时要考虑到脑转移的可能。

**图4:**
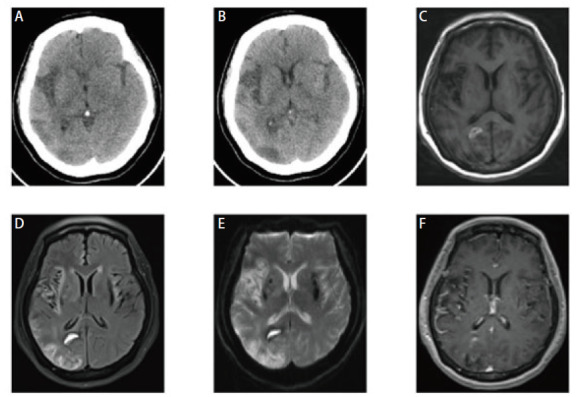
女，36岁，单纯疱疹病毒性脑炎患者。A，B：头颅CT平扫见右侧额颞顶枕叶、两侧基底节区多发低密度灶；C-F：头颅MRI增强可见右侧额颞顶枕叶、右侧背侧丘脑、双侧岛叶皮层下及左侧半卵圆中心见异常信号伴皮层略肿胀，增强扫描呈线样及沟回样强化（C：T1W低信号；D：T2 Flair高信号；E：DWI稍高信号；F：T1增强序列见线样或沟回样强化）。

**图5:**
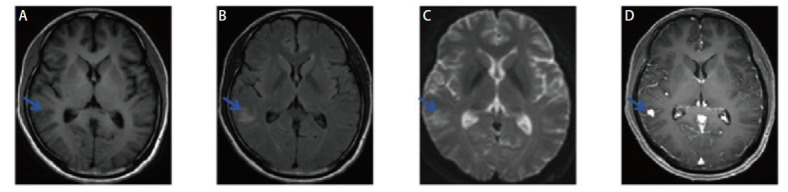
男，40岁，结核性脑膜脑炎患者。头颅MRI增强见右颞叶强化结节，结合病史，考虑结核瘤。A：T1W低信号；B：T2 Flair高信号；C：DWI未见明显异常高信号；D：T1增强序列见右颞叶结节样强化。

**图6:**
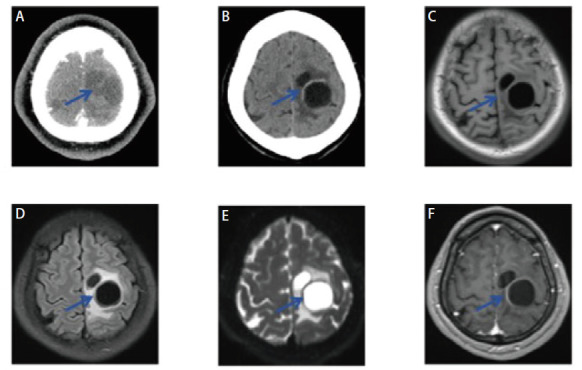
女，44岁，脑囊虫病患者。A，B：头颅CT见左侧额顶叶囊性低密度肿块，边缘见小片状钙化；C-F：头颅MRI见左侧额顶叶不规则团块状长T1长T2信号，Flair及DWI低信号，病灶局部见小结节状T1及Flair高信号影，增强扫描病灶边缘见轻度环形强化（C：T1W序列；D：T2 Flair序列；E：DWI序列；F：T1增强序列）。

**图7:**
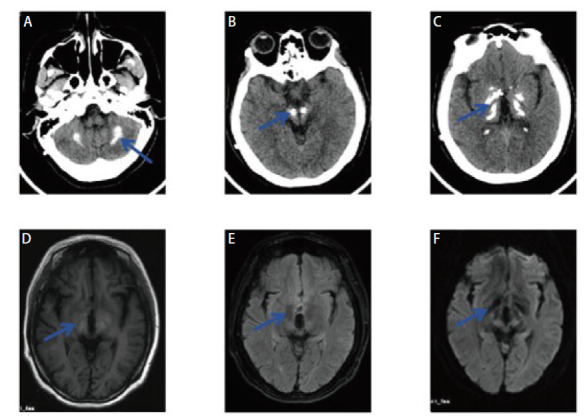
女，37岁，Fahr病患者。A-C：头颅CT见双侧基底节区、丘脑、小脑半球等多发钙化灶，对称性分布；D-F：头颅MRI见双侧基底节区、丘脑、小脑半球多发T1W高信号影（D：T1W序列；E：T2 Flair序列；F：DWI序列）。

**图8:**
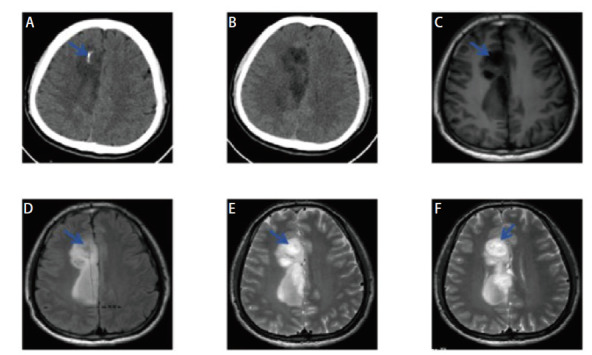
男，34岁，少突胶质细胞瘤患者。A，B：头颅CT见右侧额叶大片状低密度影，伴条状钙化灶；C-F：头颅MRI见右侧额顶叶异常信号，T1呈低信号，T2 Flair呈高信号，增强扫描呈不均匀强化（C: T1W; D: T2 Flair; E, F: 增强序列）。
